# Chemoradiotherapy with concurrent durvalumab for the palliative treatment of oligometastatic oesophageal and gastrooesophageal carcinoma with dysphagia: a single arm phase II clinical trial (PALEO, sponsored by the Australasian Gastro-Intestinal Trials Group)

**DOI:** 10.1186/s12885-022-10407-8

**Published:** 2022-12-17

**Authors:** Fiona Day, Swetha Sridharan, James Lynam, Craig Gedye, Catherine Johnson, Allison Fraser, Stephen R. Thompson, Michael Michael, Trevor Leong, Amitesh Roy, Mahesh Kumar, Andre van der Westhuizen, Gaik T. Quah, Hiren Mandaliya, Girish Mallesara, Joshua Sappiatzer, Christopher Oldmeadow, Jarad Martin

**Affiliations:** 1 Department of Medical Oncology, Calvary Mater Newcastle, Waratah, NSW Australia; 2 School of Medicine and Public Health, University of Newcastle, Callaghan, NSW Australia; 3 Hunter Medical Research Institute, New Lambton Heights, NSW Australia; 4 Department of Radiation Oncology, Calvary Mater Newcastle, Waratah, NSW Australia; 5 Prince of Wales Clinical School, Faculty of Medicine, University of New South Wales, Sydney, NSW Australia; 6 Sir Peter MacCallum Department of Oncology, Peter MacCallum Cancer Centre, University of Melbourne, Melbourne, VIC Australia; 7 Flinders Medical Centre, Bedford Park, Adelaide, SA Australia; 8 GenesisCare, Flinders Private Hospital, Bedford Park, Adelaide, SA Australia

**Keywords:** Oesophageal, Gastro-oesophageal (GOS), Dysphagia, Oligometastatic, Chemoradiotherapy, Checkpoint inhibition, Chemoradioimmunotherapy, PD-1, PD-L1, Durvalumab.

## Abstract

**Background:**

Oesophageal and gastrooesophageal junction (GOJ) carcinoma frequently present with dysphagia and de novo metastatic disease. There is scope to improve treatment paradigms to both address symptoms and improve survival. One method is integrating immune checkpoint inhibition with novel treatment combinations.

**Methods:**

PALEO is a single arm, phase II clinical trial in patients with previously untreated, oligometastatic or locoregionally advanced oesophageal or GOJ carcinoma and dysphagia. PALEO is sponsored by the Australasian Gastro-Intestinal Trials Group (AGITG). Participants receive 2 weeks of therapy with concurrent hypofractionated radiotherapy of 30Gy in 10 fractions to the primary tumour, weekly carboplatin AUC2, weekly paclitaxel 50 mg/m^2^ and durvalumab 1500 mg q4 weekly, followed by durvalumab monotherapy continuing at 1500 mg q4weekly until disease progression, unacceptable toxicity or 24 months of therapy. A single metastasis is treated with stereotactic radiotherapy of 24Gy in 3 fractions in week 7. The trial primary endpoint is the progression free survival rate at 6 months. Secondary endpoints include duration of dysphagia relief, nutritional status change, quality of life, response rate, toxicity, progression free survival and overall survival. The tertiary endpoint is prediction of outcome based on biomarkers identified from patient serial blood samples collected pre- and post-radiotherapy.

**Discussion:**

This unique investigator-initiated clinical trial is designed to simultaneously address the clinically relevant problems of dysphagia and distant disease control. The overarching aims are to improve patient nutrition, quality of life and survival with low toxicity therapy. AGITG PALEO is a multidisciplinary collaboration and will add to the understanding of the relationship between radiotherapy and the anti-tumour immune response.

**Trial registration:**

Australian and New Zealand Clinical Trials Registry: ACTRN12619001371189, registered 8 October 2019.

## Background

Oesophageal and gastro-oesophageal (GOJ) cancer are the fifth most common digestive tract cancers diagnosed in Western nations, with adenocarcinomas showing a dramatic increase in incidence since the 1970s [1]. The typical presentation is with dysphagia and weight loss. Therapeutic options for the majority of patients who present with metastatic disease include chemotherapy with or without PD-1/PD-L1 axis inhibition, radiotherapy, oesophageal stenting, or a combination of these [2], with no accepted standard of care in the context of a symptomatic primary tumour. Systemic therapy alone is not recommended for the palliation of dysphagia due to the frequency of dysphagia recurrence [3].

PALEO is an investigator-initiated Phase II clinical trial, sponsored by the Australasian Gastro-Intestinal Trials Group (AGITG), with the clinical objectives of dysphagia relief and disease control. The aim is to test the efficacy of the anti-PDL1 checkpoint inhibitor durvalumab given concurrently with chemoradiotherapy to the primary oesophageal or GOJ cancer and stereotactic radiotherapy to a single metastasis.

### Rationale for chemoradiotherapy

A phase I clinical trial conducted at our centre showed that a 30Gy in 10 fraction radiotherapy schedule given with weekly carboplatin AUC 2 and paclitaxel 50 mg/m^2^ is both tolerable and highly effective in providing dysphagia relief. Among 15 patients with metastatic oesophageal cancer and three with locally advanced disease, the median time to an improvement in dysphagia was 3 weeks, and median dysphagia free survival 5.8 months [4]. This hypofractionated radiotherapy schedule is biologically equivalent to the CROSS protocol radiotherapy dose [5], while minimizing the treatment and travel burden to the patient. The expected benefits of early dysphagia relief are the recovery of patient weight and nutrition.

### Rationale for durvalumab

The known activity of single agent PD1/PDL1 checkpoint inhibitors in later lines of palliative oesophageal and GOJ cancer treatment [6–8] has recently been augmented by demonstrated efficacy in the adjuvant setting [9] and combined with platinum-based chemotherapy in the first line metastatic setting [10, 11]. In patients with oligometastatic disease, defined as ≤5 metastases on FDG-PET scan, or locoregionally advanced and unresectable disease, we hypothesize that single agent PD1/PDL1 checkpoint inhibition will provide a superior 6 month progression free survival rate to historical survival outcomes with first line oxaliplatin / 5-fluoropyrimidine chemotherapy.

Numerous clinical trials are underway testing PD1/PDL1 inhibitors concurrent with chemoradiotherapy for oesophageal cancer, largely in the neoadjuvant setting [reviewed in Farinha et al. [12]]. The NCT03604991 Phase II/III clinical trial tested 41.4–50.4Gy radiotherapy with weekly carboplatin AUC2, paclitaxel 50 mg/m^2^ over 5 weeks either without (Arm A) or with (Arm B) concurrent nivolumab given in weeks 1 and 3. No additional toxicity was observed in the nivolumab arm in the safety run in phase [13]. For durvalumab, the NCT02962063 clinical trial in patients with resectable oesophageal or GOJ carcinomas administered 1500 mg q4weekly durvalumab concurrent with FOLFOX chemotherapy followed by 50.4Gy neoadjuvant chemoradiotherapy (chemotherapy with either carboplatin/paclitaxel or oxaliplatin/fluoropyrimidine). Those patients whom then proceed to oesophagectomy receive 6 cycles of adjuvant durvalumab 1500 mg q4 weekly. At last update from the ASCO Gastrointestinal Cancers Symposium 2021, this treatment combination was described as safe and feasible [14].

The utility of PD1/PDL1 inhibitors with chemoradiotherapy in the palliative management of obstructing oesophageal and GOJ cancers is relatively untested. A small pilot study at our centre (*n* = 5) using nivolumab concurrent with the presented hypofractionated chemoradiotherapy protocol found treatment feasible and well tolerated (unpublished data).

### Rationale for stereotactic body radiotherapy (SBRT)

The SBRT of 24Gy in 3 fractions given in this trial is for the purpose of immune priming. A substantial body of preclinical evidence suggests that radiotherapy may potentiate the anti-tumour effects of checkpoint inhibition [15]. One particularly promising avenue is via neoantigen release, resulting in T cell priming. Results in vitro suggest that this is achieved through the cGAS/STING pathway leading to increased interferon transcription [16]. This appears to be radiotherapy fraction size dependent, with ablative doses in the range of 20Gy in a single fraction inducing the Trex 1 exonuclease which inhibits this pathway. Activation appears to be optimal with 8Gy × 3, and as such, this dose delivered to a metastatic lesion has been explored in subsequent small randomized phase II studies in a range of cancers.

The Phase II PEMBRO-RT trial (*n* = 76) in non-small cell lung cancer (NSCLC) showed a significant improvement in response rate to pembrolizumab from 19 to 41% for patients randomized to 8Gy × 3 fractions to a metastatic lesion [17]. A similar NSCLC phase I/II trial (MDACC, *n* = 72) randomized patients treated with pembrolizumab to radiotherapy (50Gy in 4 fractions, or 45Gy in 15 fractions, 1–4 lesions treated), or no radiotherapy, and showed a numerical improvement in response rates and progression free survival for those treated with radiotherapy [18]. Pooled analysis of the PEMBRO-RT and MDACC trials to garner sufficient power for statistical analyses provided a cohort of 148 patients, in whom those treated with pembrolizumab and radiotherapy, versus pembrolizumab alone, derived a best overall response rate in non-irradiated lesions of 41.7% vs 19.7% (OR 2.96, 95% CI 1.46 – 6.20, *p* = 0.0039). Median progression free survival (PFS) for the radiotherapy group was 9.0 vs 4.4 months (HR 0.67, 0.45 – 0.99, *p* = 0.045) and median OS 19.2 vs 8.7 months (HR 0.69, 0.54 – 0.84, *p* = 0.0004) [19]. Early trial results such as these, and the compelling preclinical data, have prompted a recent 4-fold increase in the number of prospective clinical trials testing the combination of immunotherapy and radiotherapy [20].

Patients in the PALEO trial receive durvalumab concurrent with two radiotherapy courses: the primary tumour chemoradiotherapy in weeks 1-2, and the SBRT to a metastasis in week 7. As metastases frequently acquire new somatic mutations [21], the SBRT has the potential to increase the breadth of neoantigen exposition to T cells.

## Methods

### Aim

The aim is to determine the efficacy of durvalumab given concurrently with chemoradiotherapy to the primary oesophageal or GOJ cancer and stereotactic radiotherapy to a single metastasis. We hypothesize that the radiotherapy interventions given in PALEO will be immune priming, and hence that multimodality treatment including checkpoint inhibition will enhance disease control at all tumour sites. The ‘immune priming’ hypothesis will be tested by translational studies on serial biological samples taken before and after the respective radiotherapy courses.

### Primary endpoint

Progression free survival rate at 6 months (PFS6).

### Secondary endpoints

1. Duration of dysphagia relief (maintenance of Mellow score at least 1 point above baseline)
2. Maximum improvement in Mellow Score (ranges from 0 – normal swallow, to 4 – complete obstruction)
3. Nutritional status change as determined by:
  - Cessation of baseline enteral feeding (where relevant)
  - Patient weight
  - Patient Generated Subjective Global Assessment (PG-SGA)
4. Quality of life change (EORTC QLQ-C30 and QLQ-OES18)
5. Response rate in metastatic lesions not treated with SBRT
6. Physician graded toxicity (CTCAE v5.0)
7. Serious Adverse Event rate
8. Progression free survival
9. Overall survival
10. Exploratory endpoints:
  - Time to next systemic anti-cancer therapy
  - Immune translational studies

### Design

This is a multicentre, single arm phase II clinical trial.

### Setting

Participants will be recruited from cancer centres within Australia and New Zealand.

### Subject population

#### Target population

The target population is adults diagnosed with oligometastatic or locoregionally advanced oesophageal or GOJ carcinoma and suffering from dysphagia.

#### Inclusion criteria

1. Males and females > 18 years of age.
2. Biopsy proven adenocarcinoma or squamous cell carcinoma of the oesophagus or gastro-oesophageal junction.
3. Oligometastatic disease (1-5 lesions outside the primary tumour radiotherapy field on FDG-PET scan) or locoregionally advanced disease unsuitable for either surgical resection or radical chemoradiotherapy.
4. Symptomatic dysphagia (Mellow score > 0).
5. ECOG performance status 0-2.
6. Anticipated life expectancy of > 12 weeks.
7. Body weight of > 30 kg.
8. Adequate bone marrow function, with values within the ranges specified below. Blood transfusions are permissible.
  - White blood cell count ≥2 × 10^9^/L
  - Absolute neutrophil count ≥1.5 × 10^9^/L
  - Platelets ≥100 × 10^9^/L
  - Haemoglobin ≥90 g/L
9. Adequate liver function, with values within the ranges specified below:
  - Alanine transferase ≤2.5 x upper limit of normal (ULN)
  - Aspartate transferase ≤2.5 x ULN
  - Total bilirubin ≤1.5 x ULN (except patients with Gilbert’s Syndrome, who can have total bilirubin ≤5 x ULN)
10. Adequate renal function, with values within the ranges specified below. Note that an estimated renal function of > 125 mL/min by the Cockroft-Gault formula must not be used for carboplatin dosing, and must instead be determined using a direct method.
  - Serum creatinine ≤1.5 x ULN
  - Creatinine clearance (CrCl) ≥ 40 mL/min using Cockroft-Gault formula
11. Tumour tissue (formalin-fixed, paraffin embedded) should be available for PD-L1 and mismatch repair (MMR) protein expression and can be provided as a block or slides (archival tissue is acceptable). Blocks prepared from cytological samples, where tumour cell number is sufficient, are also acceptable. Patients will not be selected by PD-L1 or MMR status.
12. Willing and able to comply with all study requirements, including treatment, timing and/or nature of required assessments.

#### Exclusion criteria

1. Bulky or organ-threatening metastatic disease requiring upfront higher dose chemotherapy in the judgement of the treating clinician.
2. Known tumour HER2 positivity (IHC 2+ or more and HER2 gene amplification on in situ hybridisation).
3. Previous systemic therapy for oesophageal or GOJ carcinoma.
4. Previous thoracic radiotherapy. Prior palliative radiotherapy to bony metastases is permitted.
5. Oesophageal stent in situ*.*
6. Known tracheo-oesophageal fistula.
7. Known leptomeningeal or brain metastases.
8. Major surgical procedure (as defined by the Investigator) within 28 days prior to first day of study treatment. Note: Local surgery of isolated lesions for palliative intent is permitted.
9. History of another malignancy within the last 3 years, with the exception of adequately treated non-melanomatous skin cancer, carcinoma in situ and superficial transitional cell carcinoma of the bladder.
10. Prior therapy with an anti-PD1, anti-PD-L1, anti-PD-L2, anti-CTLA-4 antibody, or any other antibody or drug specifically targeting T cell co-stimulation or immune checkpoint pathways.
11. Sensory neuropathy of grade 2 or higher severity per CTCAE v5.0
12. History of allergy or hypersensitivity to study drug components, or other contraindications to any of the study drugs. Active or prior documented autoimmune disorders.
13. Any condition requiring continuous systemic treatment with either regular corticosteroids (> 10 mg daily prednisone or equivalent dose of an alternative corticosteroid) or other immunosuppressive medications within 14 days of study drug administration.
14. Positive test for hepatitis B surface antigen (HBsAg) indicating acute or chronic infection.
15. Positive test for hepatitis C virus antibody (HCV antibody), unless polymerase chain reaction is negative for HCV RNA.
16. History of other significant, or active, infection, including HIV or tuberculosis (TB).
17. Receipt of a transplanted solid organ (kidney, liver, heart or lung) or of an allogeneic bone marrow transplant.
18. Receipt of a live attenuated vaccine within 30 days prior to registration.
19. Use of alternative or traditional medicines within 14 days prior to registration.
20. Uncontrolled intercurrent illness, or psychiatric illness/social situations that would limit compliance with study requirement, substantially increase risk of incurring adverse events or compromise the ability of the patient to give written informed consent.
21. Pregnancy, lactation, or inadequate contraception.

### Treatment

**Fig. 1:**
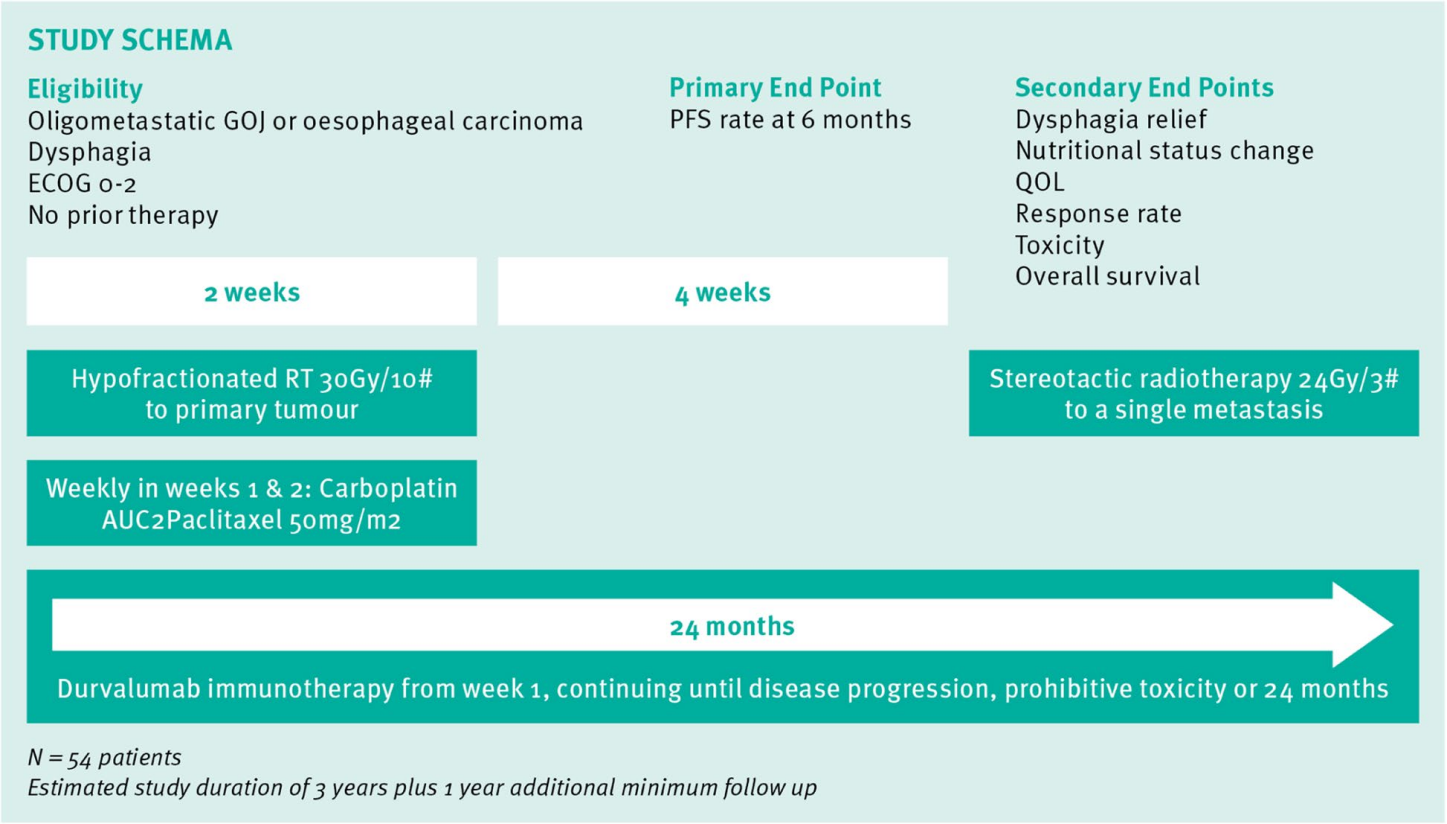
AGITG PALEO clinical trial schema

The AGITG PALEO clinical trial schema is shown in Fig. 1.

#### Chemotherapy dosing and administration

Carboplatin will be administered intravenously prior to radiotherapy on Day 1 of each of weeks 1 and 2 at a dose of area under the curve of 2 mg per millilitre per minute (AUC 2). Estimation of the participant’s glomerular filtration rate (GFR) for the purposes of carboplatin dosing will be with the Cockroft-Gault formula, unless the estimated GFR is > 125 mL/min, when direct measurement of GFR is required. The carboplatin dose must not exceed 300 mg per week. Use of pre-medications, anti-emetics and drug infusion times will be according to usual institutional practice.

Paclitaxel will be administered intravenously prior to radiotherapy on Day 1 of each of weeks 1 and 2 at a dose of 50 mg per metre square (50 mg/m^2^) of participant body surface area (BSA). Use of pre-medications, anti-emetics and drug infusion times will be according to usual institutional practice.

Dose modifications of carboplatin and paclitaxel for week 2 administration are outlined in Tables 1 and 2.

**Table 1:** Chemotherapy dose modifications for haematologic toxicity

Haematologic toxicity	Carboplatin week 2 dose	Paclitaxel week 2 dose
Absolute neutrophil count less than 1.0	Withhold	Withhold
Platelets less than 75 × 10^9^/L	Withhold	Withhold

**Table 2:** Chemotherapy dose modifications for hepatic toxicity

Hepatic toxicity	Carboplatin week 2 dose	Paclitaxel week 2 dose
Alanine transferase >3x ULN	No dose change	Withhold
Aspartate transferase >3x ULN	No dose change	Withhold
Total bilirubin > 1.5x ULN	No dose change	Withhold

#### Durvalumab dosing and administration

Durvalumab will begin on Day 1 of chemoradiotherapy and continue every 4 weeks at a dose of 1500 mg. Standard infusion time is 1 hour. If patient weight falls to ≤30 kg weight-based dosing at 20 mg/kg will be administered using an IV bag size selected such that the final concentration is within 1 to 15 mg/mL.

No dose modifications are permitted for durvalumab. Delayed doses that fall outside the cycle treatment window (+ 3 days) will not be replaced per a ‘time marches on’ policy.

Durvalumab will continue until disease progression by iRECIST assessment, unacceptable toxicity, patient withdrawal or 24 months.

#### Treatment beyond progression

Accumulating evidence indicates a minority of participants treated with immunotherapy may derive clinical benefit despite initial evidence of progressive disease (PD). Participants treated with durvalumab will be permitted to continue treatment beyond iRECIST defined PD (iCPD) up to a maximum of 24 months from date of first dose as long as they meet the following criteria:

- Investigator-assessed clinical benefit.
- Tolerance of durvalumab.
- Stable performance status.
- Treatment beyond progression will not delay an imminent intervention to prevent serious complications of disease progression (e.g. CNS metastases).
- Radiographic assessments with CT scan of chest / abdomen and pelvis should continue at 6 weekly intervals for two more CT studies after iCPD before moving to 12 weekly imaging per the Schedule of Assessments for patients otherwise after Week 25.

For the participants who continue durvalumab beyond progression, further disease progression is defined as an additional 10% increase in tumour burden with a minimum 5 mm absolute increase from the time of iCPD. This includes an increase in the sum of diameters of all target lesions and/or the diameters of new measurable lesions compared to the time of iCPD. Treatment should be discontinued permanently upon documentation of further progression.

New lesions are considered measurable at the time of progression if the longest diameter is at least 10 mm (except for pathological lymph nodes which must have a short axis of at least 15 mm). Any new lesion considered non-measurable at the time of initial progression may become measurable and therefore included in the tumour burden if the longest diameter increases to at least 10 mm (except for pathological lymph nodes which must have a short axis of at least 15 mm). In situations where the relative increase in total tumor burden by 10% is solely due to inclusion of new lesions which become measurable, these new lesions must demonstrate an absolute increase of at least 5 mm.

#### Primary tumour radiotherapy

This will follow institutional protocols, which need to be centrally reviewed during the credentialing of a centre. General principles are outlined below.

- GTV (Gross Tumour Volume) – Gross primary disease evident on endoscopy as well as other modalities including CT, PET and MRI. Adjacent nodal disease can be included if encompassable within radiation portal but is not mandatory.
- CTV (Clinical Target Volume) – GTV or ITV + 0-1 cm superiorly and inferiorly and 0-1 cm radially. Superior and inferior extent can be modified at investigator discretion, particularly if concerns regarding radiation dose to the lung.
- PTV (Planning Target Volume) – CTV + 0.7 cm in all directions.

Radiation therapy will be delivered to the primary disease to a dose of 30Gy in 10 fractions at 3Gy per fraction, over 10 consecutive working days. Weekly chemotherapy (carboplatin AUC 2/paclitaxel 50 mg/m^2^) to be delivered on Day 1 (+ 1) and Day 8 (+ 1) of radiation therapy. Durvalumab 1500 mg should be delivered on Day 1 (+ 1) of radiation therapy. Planning technique – 3D conformal radiotherapy, Intensity Modulated Radiotherapy (IMRT) or Volumetric Modulated Arc Therapy (VMAT) may be used. PTV D98 to be covered by at least 95% of the prescribed dose (28.5Gy).

#### Stereotactic body radiotherapy (SBRT) to a single metastatic site

A single metastasis will be treated with stereotactic radiotherapy (24Gy in 3 fractions) 4 weeks after the completion of the chemoradiotherapy to the primary tumour. When selecting a lesion for stereotactic treatment the lesion should be at least 10 mm in size in the short axis for the purposes of delineation and localisation at the time of treatment. If more than one lesion can be treated, the treating radiation oncologist has the discretion to identify which lesion should be treated. This may be an FDG-PET avid lymph node, lung, adrenal or liver lesion. Bony metastases can be targeted, but may be less immunogenic, and hence the preference is to treat a soft tissue lesion. The metastasis being treated must not have received significant dose (i.e. must have Dmax<3Gy) in the previously irradiated primary tumour field. Similarly, if an organ at risk can receive dose from both the CRT and SBRT, this must also be considered in both lesion selection as well as organ tolerance. Patients in whom a suitable metastasis for SBRT cannot be identified, but otherwise meet PALEO eligibility criteria, should be discussed with the Radiation Oncology Study Chair.

Within the centre, all SBRT contours must undergo peer review by another radiation oncologist familiar with stereotactic radiotherapy treatments. In challenging cases, the local investigator is encouraged to contact the trial Radiation Oncology Study Chair to discuss.

Dose prescribing and recording should be according to ICRU 83 [22]. Radiation dose of 24Gy in 3 fractions at 8Gy per fraction to be delivered on alternate days at least 48 hours apart over a maximum of 7 days. Table 3 shows the organ at risk (OAR) radiotherapy dose constraints.

**Table 3:** DVH constraints for OAR [23]

Organ	Parameter	Dose-Volume Constraints
Spinal cord PRV	D Max 0.1 cc	0.1 cc < 18Gy
Kidney	Maximum dose/volume Dose > 200 cc If total kidney volume < 200 cc or treating adrenal lesion ensure contralateral kidney dose kept to minimum	< 16Gy
Heart	D max 0.5 cc	<24Gy
Small Bowel	D max 0.5 cc D 5 cc	< 22.2Gy < 16.5Gy
Rectum	D max 0.5 cc	< 28.2Gy
Stomach	D max 0.5 cc D 10 cc	< 22.2Gy < 16.5Gy
Skin	D max 0.1 cc D 10 cc	<33Gy <20Gy
Lungs - GTV	V20Gy V12.5Gy	< 10% < 15%
Liver	D > 700 cc	<15Gy
Large Bowel	D max 0.5 cc	< 28.2Gy

**Fig. 2:**
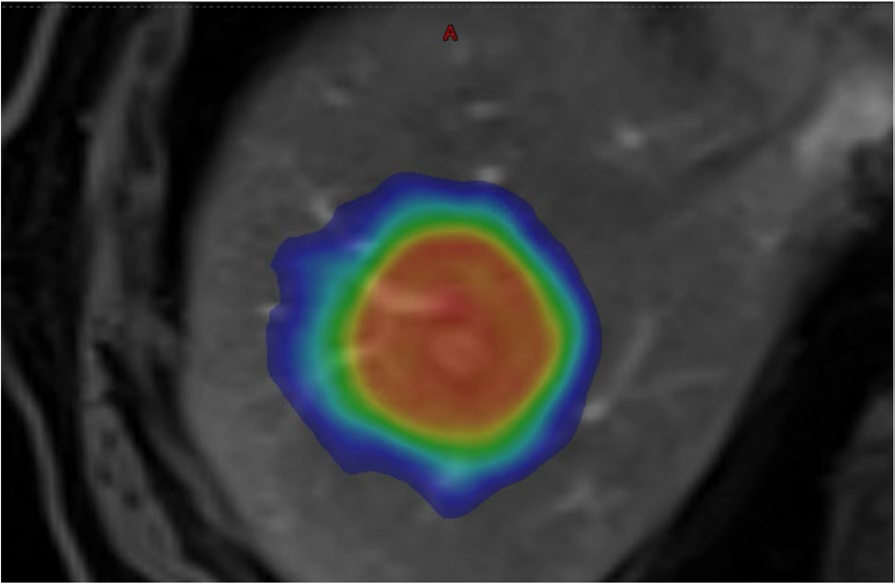
Planning MRI image of SBRT (24Gy / 3#) dose wash to a liver metastasis (image T1 weighted, fat saturated, post-liver specific contrast in the arterial phase)

An example of a radiotherapy dose wash of a liver metastasis treated with SBRT is shown in Fig. 2. Durvalumab will continue to be delivered q4weekly during radiation as per the study protocol.

### Statistical analysis

#### Sample size

While there is no accepted standard of care for the patient population eligible for the AGITG PALEO trial, in patients with metastatic GOJ or gastric cancer and any burden of disease, the 6 month PFS rate is approximately 50% for first line oxaliplatin and infusional 5-Fluorouracil chemotherapy [24]. To rule out an unacceptable similar 6 month PFS rate of 50% in favour of 67.5%, with 80% power and a one-sided 0.05 significance level, 51 patients would be needed. A PFS6 of 67.5% is also deemed to be of clinical utility and to warrant further testing of this protocol in a Phase III randomized clinical trial. To account for an estimated 5% patient dropout rate, the sample size for PALEO is 54 patients.

#### Outcomes

The primary measure of effect for this trial is the progression free survival rate at 6 months (PFS6). The 6 month PFS rate and its 95% confidence interval will be determined using the Kaplan Meier method.

The secondary outcome measures of progression free survival and overall survival will be reported with Kaplan Meier curves and summarized by their medians, and proportions event-free at 1 year. The objective tumour response rate will be reported as binomial proportions on the evaluable patient subgroup with 95% confidence intervals.

Analyses of safety endpoints will include all patients who received at least one radiotherapy fraction and/or one dose of carboplatin, paclitaxel or durvalumab. Reporting of safety endpoints will include summaries of adverse events experienced whilst on treatment and to 90 days after the last durvalumab dose, and their grades.

## Discussion

Individual patients with metastatic oesophageal and GOJ cancers vary in the respective burdens posed by their metastatic disease and the primary tumour. For those with organ-threatening metastatic disease, upfront platinum-based therapy with/without concurrent checkpoint inhibition depending on their tumour PDL1 combined positive score [10, 11], may be most appropriate. Conversely patients with low volume distant disease but a symptomatic primary tumour may instead be considered for local therapies, or multimodal treatment, after multidisciplinary discussion.

The population eligible for the PALEO clinical trial has been carefully selected as those patients most likely to benefit from combined modality therapy. The PALEO protocol delivers upfront local treatment to the primary tumour for early symptomatic and nutritional benefit, supported by our existing data shows that a 2 week chemoradiotherapy protocol (30Gy/10# with concurrent carboplatin and paclitaxel) provides rapid dysphagia relief [4]. Patients must have low volume metastatic disease (≤5 metastases, non-bulky) based on evidence that the ratio between activated T cells and tumour burden is critical for immune checkpoint inhibitor effect [23, 25, 26]. Patients are eligible for participation with ECOG performance status 0-2 as many patients have experienced substantial weight loss at oesophageal cancer diagnosis, and single agent PD1/PDL1 inhibition is better tolerated than platinum-based chemotherapy [27].

Participant enrolment to AGITG PALEO is independent of tumour biomarkers PDL1 expression and mismatch repair deficiency. Results from the treatment of NSCLC suggest that the clinical benefit gained from concurrent or sequenced radiotherapy and checkpoint inhibition is biomarker agnostic. While the anti-PD1 immunotherapy response in metastatic NSCLC is heavily influenced by tumour PD-L1 expression [28, 29], in the PACIFIC trial in stage III NSCLC the benefit of 12 months durvalumab immediately following chemoradiotherapy was irrespective of baseline tumour PD-L1 expression [30]. In the PEMBRO-RT trial in NSCLC, the benefit of radiotherapy given concurrent with single agent pembrolizumab was largest in the patient subgroup with PDL1 negative tumours.

The PALEO primary endpoint of PFS6 has been proposed as the most reliable surrogate for overall survival in immune checkpoint inhibitor trials, and hence for identifying single arm phase 2 clinical trial protocols to consider for future randomized studies [31]. A recent systematic review and meta-analysis confirmed PFS6 as a reliable surrogate for OS [32]. In this trial, PFS6 captures disease control at all tumour sites (primary and metastases) and hence also reflects ongoing dysphagia control and the deferral of alternate systemic therapy. The secondary trial endpoints specifically measure swallowing, nutritional and quality of life outcomes.

The most important translational focus of the PALEO trial is the humoral and cellular effects of the two radiotherapy courses given with concurrent durvalumab. Serial blood samples are collected at baseline and pre- and post- the respective radiotherapy treatments, at the time of confirmed disease progression, and in the event of any grade 3 or higher immune-mediated adverse event. To test the ‘immune priming’ hypothesis of radiotherapy and checkpoint inhibitor interaction, blood samples will be tested for T cell receptor diversity for comparative profiling of the immune repertoire at baseline and post-therapy in a subset of patients deemed excellent treatment responders, or non-responders. Other planned exploratory blood analyses include plasma whole proteome analysis and RNAseq for measurement of changes in gene expression and cell signaling pathways post-radiotherapy. Tumour diagnostic biopsies are also collected in the PALEO trial for correlative tissue-based translational studies.

## Data Availability

Not applicable. No data reported.

## Abbreviations

AGITG: Australasian Gastro-Intestinal Trials Group
ASCO: American Society of Clinical Oncology
AUC: Area Under the Curve
BSA: Body Surface Area
CNS: Central Nervous System
CPS: Combined Positive Score
CRT: Chemoradiotherapy
CT: Computed Tomography
CTCAE: Common Terminology Criteria for Adverse Events
CTLA4: Cytotoxic T-Lymphocyte Associated protein 4
CTV: Clinical Target Volume
DVH: Dose-Volume Histogram
FDG: Fluorodeoxyglucose
GFR: Glomerular Filtration Rate
GOJ: Gastro-oesophageal junction
GTV: Gross Tumour Volume
Gy: Gray
iCPD: Confirmed Progressive Disease (iRECIST)
ICRU: International Commission on Radiation Units and Measurements
IMRT: Intensity Modulated Radiotherapy
iRECIST: Immune Response Evaluation Criteria In Solid Tumours
ITV: Internal Target Volume
iUPD: Unconfirmed Progressive Disease (iRECIST)
MMR: Mismatch Repair
MRI: Magnetic Resonance Imaging
NSCLC: Non Small Cell Lung Cancer
OAR: Organs At Risk
OS: Overall Survival
PALEO: Phase II clinical trial of chemoradioimmunotherapy for the ALleviation of oEsOphageal cancer complications
PD1: Programmed Death 1
PDL1: Programmed Death Ligand 1
PET: Positron Emission Tomography
PFS: Progression Free Survival
PFS6: Progression Free Survival rate at 6 months
PTV: Planning Target Volume
RCT: Randomised Controlled Trial
RECIST: Response Evaluation Criteria In Solid Tumours
SBRT: Stereotactic Body Radiotherapy
VMAT: Volumetric Modulated Arc Therapy
#: Radiotherapy fraction
